# Ischemia of the Penis and Fingertips Secondary to Calcifying Uremic Arteriolopathy

**DOI:** 10.7759/cureus.7481

**Published:** 2020-03-31

**Authors:** Hatim Jroundi, Wiame El Alj, Mihai Razvan Manescu, Yassamine Bentata, Moulay Hassan Farih

**Affiliations:** 1 Urology, Hassan II University Hospital Center/Sidi Mohammed Ben Abdellah University, Fez, MAR; 2 Nephrology, Mohammed VI University Hospital Center/Mohammed First University, Oujda, MAR; 3 Urology, Hauts-Clos Hospital, Troyes, FRA

**Keywords:** calcific uremic arteriolopathy, penile arteries, ischemia of the penis, chronic renal failure

## Abstract

Calcifying uremic arteriolopathy (CUA), also called calciphylaxis, refers to the calcification of the walls of the arteries of medium and small caliber, causing ischemic skin lesions. Diagnosis should be made if ischemic lesion develops in a patient with chronic renal failure (CRF), and it is confirmed based on clinical, radiological, and histological criteria. Generalized CUA characterized by ischemia of the penis (IP) along with other localizations of cutaneous ischemia is exceptional, and the morbidity and high mortality rate associated with this entity most often warrant multidisciplinary and conservative management.

## Introduction

Calcifying uremic arteriolopathy (CUA) refers to the obstruction of small- and medium-caliber vessels by calcifications resulting in ischemic lesions; it is a disease that primarily affects patients with chronic renal failure (CRF) irrespective of whether they are receiving dialysis or not [1]. The penile localization of this disease is rare, and the generalized form characterized by ischemia of the penis (IP) along with other cutaneous ischemic localizations is exceptional and linked to high mortality, often requiring multidisciplinary and conservative management [2]. We present the clinical case of a bifocal CUA presenting with IP and ischemia of the fingertips in a 78-year-old man suffering from chronic CRF secondary to diabetic nephropathy. He underwent conservative treatment and died six days later due to hemodynamic instability.

## Case presentation

**Figure 1:**
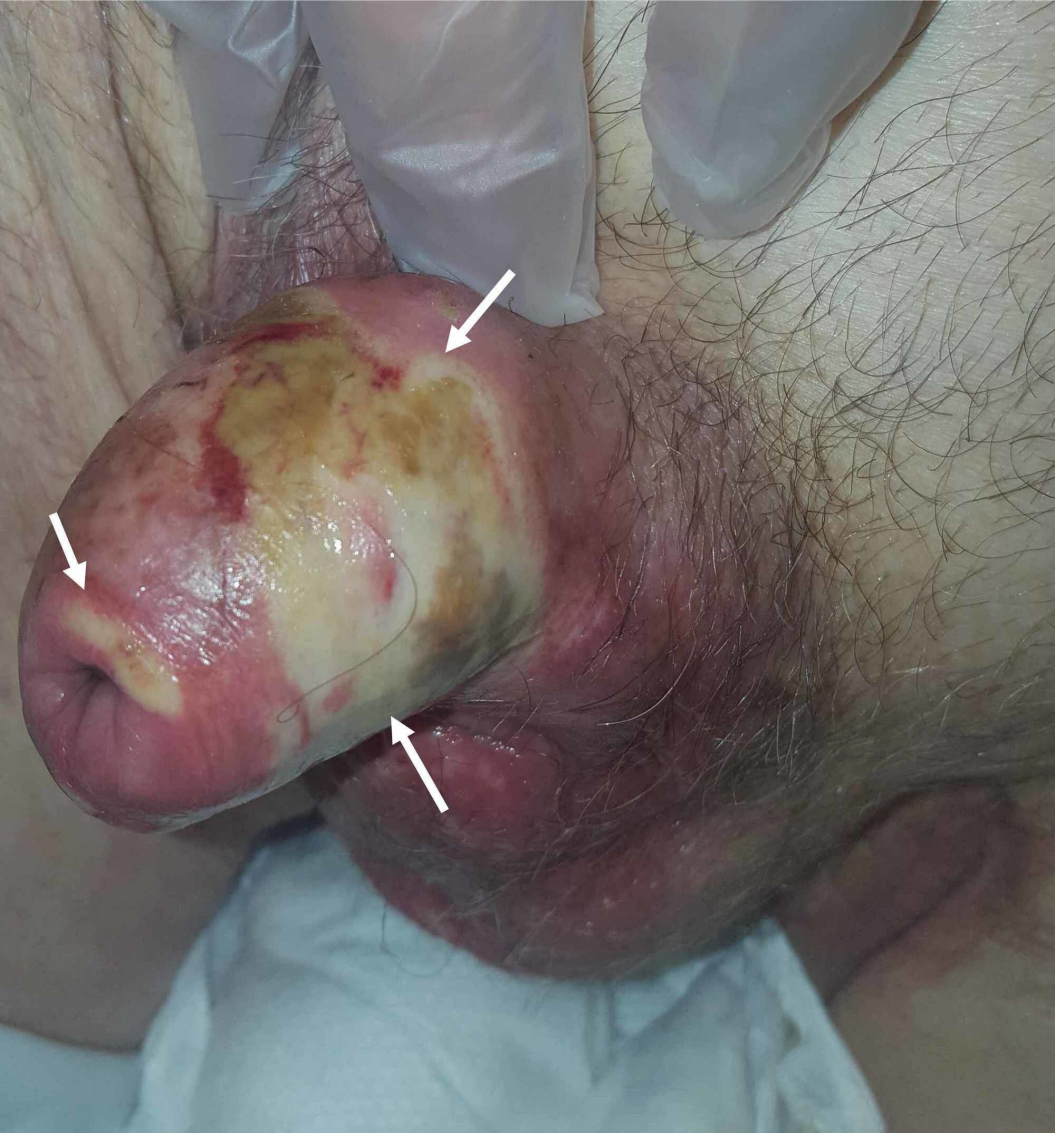
Examination of the external genitalia The image shows phimosis associated with areas of extensive ischemia on the penile and scrotal wall (white arrows)

**Figure 2:**
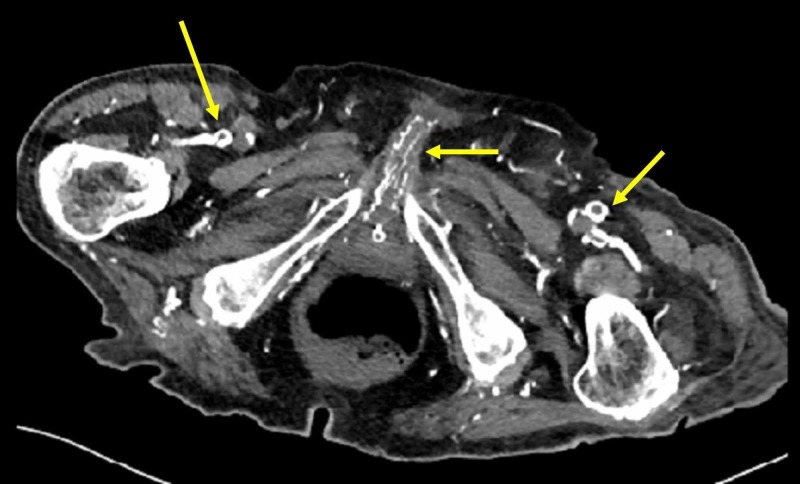
Axial view of the abdominal/pelvic CT scan The image shows diffuse calcifications of the femoral arteries and their branches of division and penile arteries tributary to the internal pudendal artery (yellow arrows) CT: computed tomography

A 78-year-old man was admitted to the emergency department for penile pain that had evolved over the past two days. His history was remarkable for CRF secondary to diabetic nephropathy, bilateral iliac angioplasty for atheromatous disease, and hypertension. On physical examination, the patient was conscious, hemodynamically stable, and afebrile, with the presence of a full bladder. Examination of the external genitalia showed phimosis associated with areas of extensive ischemia on the penile and scrotal wall (Figure 1). The blood test showed leukocytes at 12,400/mm^3^, C-reactive protein at 302 mg/l (normal: <6 mg/l), glomerular filtration rate at 28 ml/min/1.73m2, normal blood glucose, corrected blood calcium at 9 mg/dl, blood phosphorus at 8 mg/dl, blood vitamin D at 5 ng/ml, and parathyroid hormone at 300 pg/ml. All bacteriological samples were negative. An abdominal/pelvic CT scan showed diffused calcifications of the abdominal aorta, iliac arteries, femoral arteries, and peripheral arteries, including the deep arteries of the penis (Figure 2).

**Figure 3:**
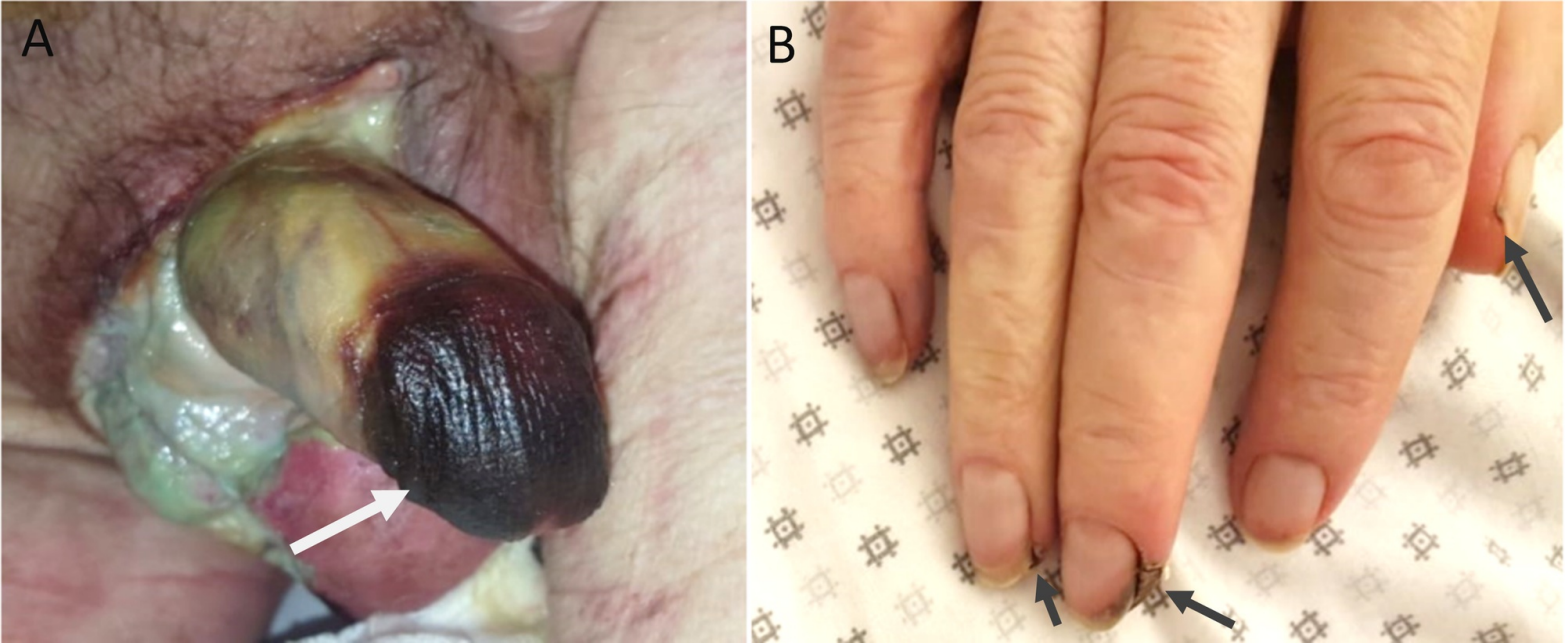
Photos after four days of clinical treatment A: glans necrosis (white arrow); B: necrosis of the fingertips

Surgical treatment consisted of performing a posthectomy that involved debridement of the wall of the penis and the placement of a suprapubic catheter. An anatomicopathological examination of the foreskin and scrotal skin had shown areas of necrosis with micro-calcifications of the vascular walls. The patient was put on medical treatment, including analgesics, cinacalcet, and vitamin D supplementation, with daily dressing changes. The evolution was marked by the appearance of necrosis of the glans and the fingertips four days later (Figure 3). On the sixth day post-surgery, the patient expired due to hemodynamic instability.

## Discussion

CUA is a rare vascular disease responsible for cutaneous ischemic events, but can also be systemic [1]. It is a disease that primarily affects patients with CRF irrespective of whether they are receiving dialysis or not [3,4]. Also called calciphylaxis, its main risk factors are phosphocalcic metabolism disorders, especially a high calcium-phosphorus product, obesity, the female sex, states of hypercoagulability, and some autoimmune diseases [5]. In our patient, phosphocalcic metabolism disorders were probably the main factors involved in the onset of this disease.

Despite the richness of penile vascularization, the obliteration of the penile arteries by calcium deposits secondary to a CUA can lead to IP. The latter is entirely different from Fournier's gangrene, which has an infectious origin. Penile pain is often present and can sometimes precede the appearance of skin lesions [6]. Imaging can help diagnose the disease. Doppler ultrasound makes it possible to assess the permeability of the penile arteries; the pelvic and abdominal CT scan shows pelvic vascular calcification, and MRI studies can show the extent of ischemic lesions [7].

Histological examination of skin biopsies can confirm the diagnosis by showing calcifications of the vessels associated with skin necrosis [8]. IP secondary to a CUA and associated with other skin localizations have been described in some studies [9,10]. Penile CUA is considered a sign of an inferior prognosis [11]. This localization is associated with a high mortality rate, hence justifying conservative management at first [2].

The conservative treatment must be quick, and it includes a posthectomy in which a suprapubic catheter is placed to facilitate the observation of the glans and to ensure dry scarring. Aggressive treatment is recommended in the event of gangrene progression or in patients without severe comorbidities; it includes a total or partial penectomy [12]. In the case of associated CRF, medical management involves correcting the phosphocalcic balance (which can go as far as surgical parathyroidectomy in the event of failure of medical treatment), optimizing dialysis, and the intravenous administration of sodium thiosulfate [8,13,14]. This last treatment is administered three times a week at a dose of 25 g at the last hour of hemodialysis [11,12]. In patients not on dialysis, the dose will be adapted based on renal function [15]. Despite adequate management, mortality is around 80% in patients with renal failure in cases of cutaneous ischemia alone [16]. It is most often secondary to infectious complications.

## Conclusions

IP associated with other ischemic localizations of the skin in the context of CUA is a rare and severe entity. It occurs mainly in patients with CRF, whether they are on dialysis or not. Therapeutic management is multidisciplinary and depends mainly on the general condition and comorbidities of the patient. The prognosis is generally poor, with a very high mortality rate.
